# Systems pharmacology approach to investigate the mechanism of *Artemisia argyi* in treating rheumatic diseases

**DOI:** 10.1038/s41598-022-23635-6

**Published:** 2022-11-05

**Authors:** Yuanzhi Huang, Xupeng Jin, Jiayi Liu, Wei Wu, Huiping Wang

**Affiliations:** 1 Hubei Key Laboratory of Economic Forest Germplasm Improvement and Resources Comprehensive Utilization, Hubei Collaborative Innovation Center for the Characteristic Resources Exploitation of Dabie Mountains, Huanggang Normal University, Huangzhou, 438000 China; 2 Hanchuan Maternal and Child Health Hospital of Hubei Province, Hanchuan, 431600 Hubei China

**Keywords:** Computational biology and bioinformatics, Medical research

## Abstract

*Artemisia argyi* (AA) has been proven to be effective in the adjuvant treatment of rheumatism (RA), but the mechanism of its action in RA is not clear. This study aims to clarify the molecular mechanism of AA as a potential therapy for RA by using network pharmacology. The TCM systems pharmacology (TCMSP) was used to screen the active components of AA, and identification of the potential target genes of active compounds and rheumatism was performed with PharmMapper and GeneCards, respectively. Construction of complex target networks and protein–protein interaction networks was based on the Cytoscape software. The biological functions and pathway analysis of targets and effective targets were analyzed using DAVID. Our study demonstrated that 105 target genes were associated with these active compounds and RA. ALB, AKT1, and MAPK1 were the first three hub genes, and the metabolic and signaling pathways related to these hub genes were remarkably abundant. Results showed that AA might play a role in RA by affecting multiple targets and multiple ways, reflecting that TCM was characterized by multicomponents and multitargets. AA has the potential to be a promising new candidate for the treatment of RA and has value for further research and development.

## Introduction

Rheumatism is a kind of disease that predominantly affects bone, joint, synovial bursa, tendon, fascia, muscle, nerve and other surrounding soft tissue. The prevalence of rheumatic diseases ranges from 11.6% to 46.4%, depending on the region, study protocol, and age of respondents. The prevalence of symptomatic osteoarthritis ranges from 5.1% to 20.8%, with the lumbar spine, knees, and cervical spine as the most affecte^1^. The disease is complex, lingering, and difficult to recover and has a high disability rate, thereby seriously threatening the physical and mental health of human beings. The main drugs used to treat rheumatoid arthritis (RA) usually have significant side effects^2,3^. Moxibustion is considered a safe and effective way to treat RA^4^.

Moxibustion, a traditional Chinese therapy, has been used for thousands of years in China and its neighbor^5^. In clinical practice, moxibustion therapy is usually used to treat RA and includes traditional (pure moxa stick) or indirect moxibustion methods, which are performed by implanting insulation materials between the acupoints and moxa stick^6–8^. Moxibustion can improve the immune function of the body through anti-inflammatory and immune effects, inhibit the secretion of synovial cytokines, and play a therapeutic role in rheumatoid arthritis^9^. Referring to the theory of TCM, moxibustion can help to warm and activate meridians and promote blood circulation and qi^5^. The results of randomized clinical trials by meta-analysis showed that the combination of moxibustion and drug therapy has a favorable effect in terms of effectiveness compared with conventional drug therapy alone^10^. Researchers analyzed the efficacy and safety of moxibustion in the treatment of knee osteoarthritis and showed that moxibustion has significant curative effect compared with Western medicine^11^. The clinical and experimental studies have confirmed that moxibustion may relieve the symptoms of osteoporosis, which can be explained that regulation of the bone metabolism index and endocrine and protein expression levels will help to reach the steady state of bone metabolism and inhibit osteoclast activity, thereby reducing bone resorption^12,13^. All the above studies supported that moxibustion is effective in the treatment of RA.

The leaves of *Artemisia argyi* (AA) are a common Chinese herbal medicine, which can be used as medicine or food, and have dual value of medicine and food. They are widely used to produce moxa stick as the main raw material in China^5,14^. In the moxibustion treatment, the antioxidants in AA combustion products adhere to the skin at acupoints and penetrate the body through the heat of moxibustion. Therefore, AA, as the raw material of moxibustion, plays a key role in the treatment of RA. However, the pharmacological mechanism of AA is still unclear, and no relevant research is available, which affects its clinical application and the research and development of related new drugs^15^. Network pharmacology based on the theory of system biology, whose systematic and multi-dimensional analysis characteristics are consistent with the holistic view of TCM, provides a powerful tool for revealing the complex mechanism of TCM. The research shows that the trend of combining calculation, experiment and clinic is a promising development direction of TCM network pharmacology^16^. In our study, network pharmacology method was used to predict and analyze the mechanism of AA in the treatment of RA from the molecular level, so as to provide scientific basis for the clinical application of AA and the development of new drugs.

## Materials and methods

### Screening for active components of AA

The principal components of AA were referred to the TCM systems pharmacology (TCMSP) database and analysis platfor^17^.

Oral bioavailability (OB) is a major index that reflects the degree to which oral drugs reach the targets by passing through intestinal wall barriers. Compounds with low OB may exhibit minimal effect because few active components appear in the blood. The drug-likeness (DL) index is applied in the identification of drugs and nondrugs, and compounds with low DL values may not be drugs^18^. Brain capillary wall and glial cells formed a barrier between plasma and brain cells, and so do choroid plexus formed between plasma and cerebrospinal fluid, which are called the blood–brain barrier (BBB), preventing certain substances (mainly harmful) from entering brain tissue through blood. The permeability of BBB is an indicator of drug permeability. In our research, 30%, 0.18, and 0.3 were selected as the best cutoff values of OB, DL, and BBB, respectively^19,20^. The components of AA with values higher than these reference standards were regarded as functional constituents.

### Prediction of target genes

To investigate the relation between each selected active compound and its target genes, the main active ingredients of AA were organized in SDF and MOL2 formats, and then uploaded to the PharmMapper server (http://59.78.98.102/pharmmapper/) and analyze^21–23^. Using “human protein targets are only used to select target sets” as the search term, the rest were set by default. The selected protein target was imported into the UniProt database (https://www.uniprot.org/) in PDB ID format. The prediction indexes of the effective components of AA were achieved by searching and converting.

### Screening of rheumatic disease targets

The keyword “rheumatic diseases” was used to search GeneCards (https://www.genecards.org/) to obtain the reported genes associated with rheumatic diseases.

### Network construction and analysis

The target information of effective components in AA and disease target genes was processed with the Cytoscape 3.8 software (http://cytoscape.org/), and used to construct the model of the medicine-ingredients-disease-targets (M-I-D-T) network. In this network model, nodes represented molecules or target proteins, whereas edges represented relationships among components, disease, and targets.

### Establishment of the protein–protein interaction network

The Search Tool for the Retrieval of Interacting Genes (STRING) database is an effective tool for exploring and constructing protein–protein interaction (PPI) networks^24^. By using the online STRING tool (version 11.0), the PPI of target genes was picked out while a required interaction score was above 0.4, and the target genes of PPI network having high connectivity degrees were selected as hub genes.

### GO and KEGG pathway analysis

The biological functions of these selected genes were analyzed using the Database for Annotation, Visualization and Integrated Discovery (DAVID), so do pathway analysis of target biological functions and effective targets. Data were exported from David and screened for count > 5, p-value < 0.05, and FDR < 0.05. By using Cytoscape, a compound–target–pathway network was set up to understand the relationship among RA, targets, and compounds systematically.

### Molecular docking

The 3D structure of key compounds was made by Chem Office software, then the 3D structure of core protein gene was downloaded from PDB database, the protein was dehydrated and dephosphorized by PyMOL software, the pdb format of compound and core protein gene was converted into pdbqt format and active pocket was found by AutoDock 1.5.6 software, and finally, Vina was run for docking. The binding activity of the two is evaluated according to the binding energy, and the binding energy ≤ − 5.0 kJ/mol is selected as the screening basis to evaluate the reliability of network analysis and prediction.

## Results

### Screening of active compounds

By considering OB ≥ 30%, DL ≥ 0.18, and BBB ≥ 0.3, TCMSP results showed 135 traditional components in AA, among which seven active compounds were retrieved from TCMSP (Table 1). The PharmMapper integrated pharmacophore matching platform was used for reverse prediction of these active compounds.

**Table 1:** Active compounds of AA.

ID	Molecule name	MW	OB (%)	BBB	DL
MOL001494	Mandenol	308.56	42	1.14	0.19
MOL002883	Ethyl oleate (NF)	310.58	32.4	1.1	0.19
MOL000358	Beta-sitosterol	414.79	36.91	0.99	0.75
MOL000449	Stigmasterol	412.77	43.83	1	0.76
MOL005720	24-methylenecyloartanone	438.81	41.11	1.26	0.79
MOL005735	Dammaradienyl acetate	454.81	44.83	1.07	0.83
MOL005741	Cycloartenol acetate	468.84	41.11	1.33	0.8

### Target gene prediction

**Figure 1:**
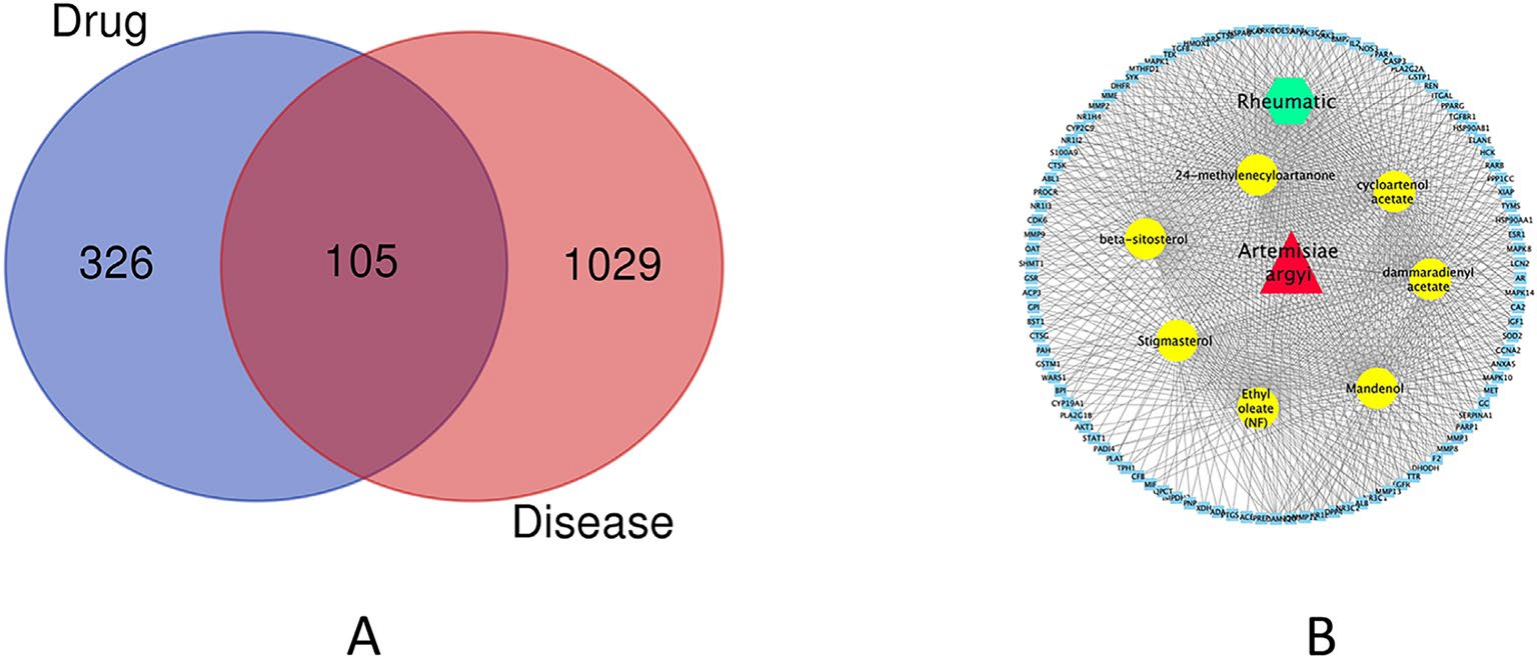
Construction of the compound-disease network. (**A**) Venn diagram of target genes for RA and active compounds. RA has 1134 target genes, whereas active components have 431 target genes. A total of 105 overlapping target genes are found between the two sets, (**B**) Medicine-ingredient-target-disease network with four parts (Red: *Artemisia argyi* (AA), green: RA, yellow: active ingredients of AA, blue: 105 potential common targets) was generated by Cytoscape (Version, 3.8) (http://cytoscape.org/).

We identified 1134 target genes corresponding to RA from GeneCards and 431 target genes for active compounds from the Uniprot identifiers map after elimination of duplicates. On the basis of the above results, 105 overlapping genes between RA and active components were obtained (Fig. 1A).

### M-I-D-T network

Through the treatment of the software of Cytoscape, a M-I-D-T network was produced and showed that the four components were closely related to one another (Fig. 1B).

### PPI network

**Figure 2:**
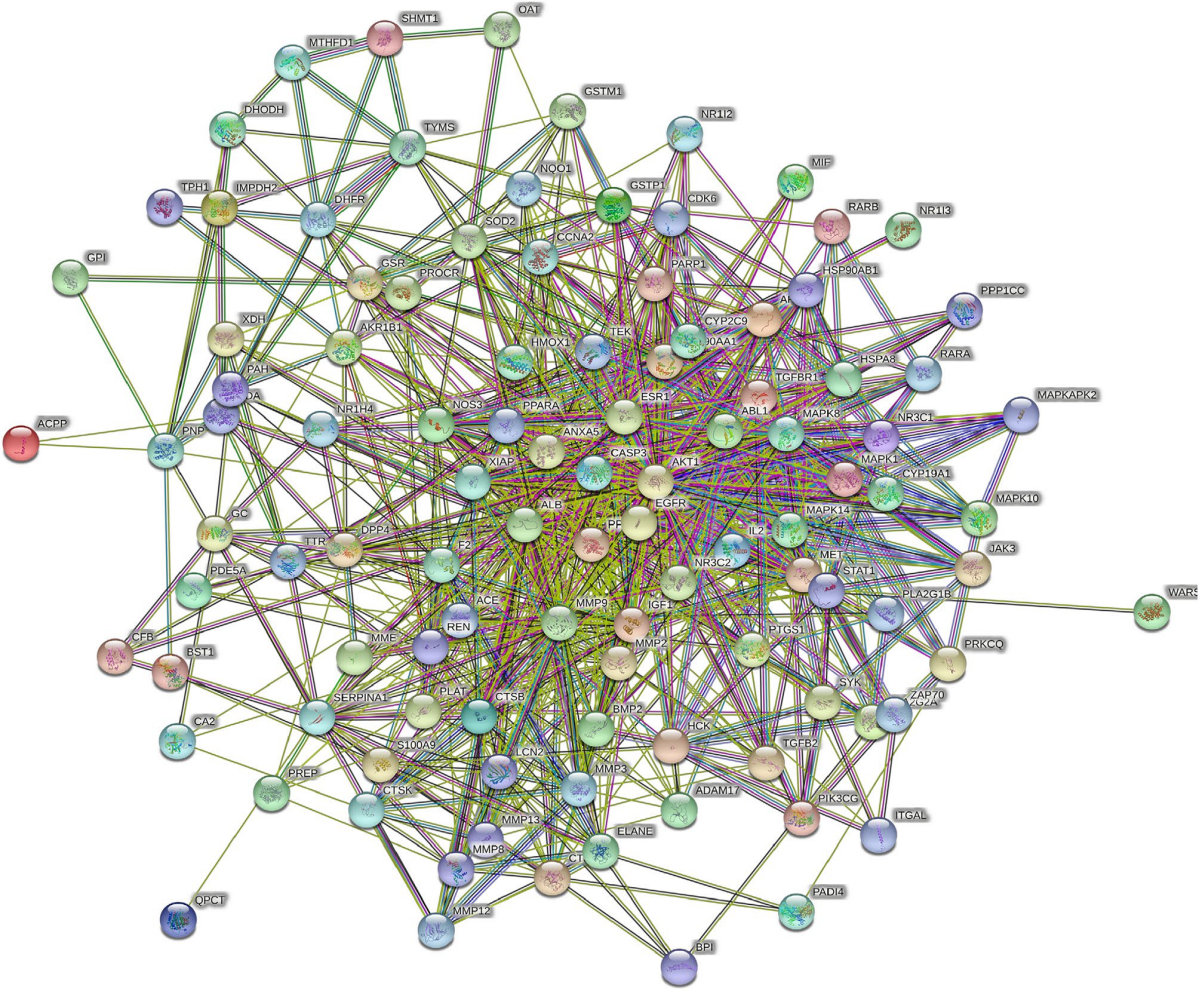
Protein–protein interaction (PPI) network analysis of 105 potential targets was generated by String (Version, 11.5) (https://cn.string-db.org/).

**Figure 3:**
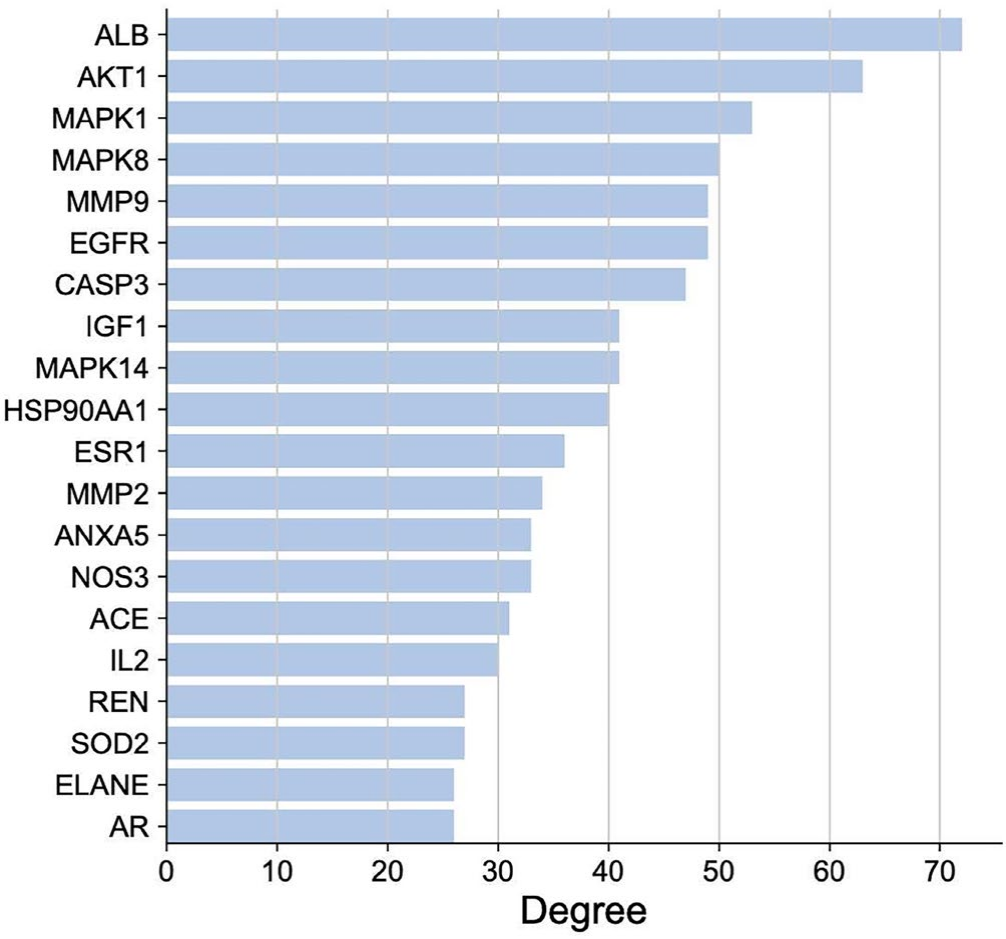
Top 20 targets from the PPI network.

On the basis of the STRING database, we generated a PPI network containing 105 overlapping target genes (Fig. 2). Each protein was represented by a node, and the interaction was represented by lines between nodes. The number of lines connected to a specified node was considered as the connectivity degree. In the present research, hub genes were defined as genes with connectivity degree > 5. As shown in Fig. 3, among 20 hub genes, ALB, AKT1, MAPK1, MAPK8, MMP9, EGFR, CASP3, and IGF1 were the top 8 hub genes.

### GO and KEGG pathway enrichment analyses

Based on the GO enrichment analysis, major targets could be separated into various functional modules, and the GO enrichment analysis was performed using the DAVID ver 6.8 gene annotation platform. Data were exported from the DAVID database, and the data of biological process (BP), molecular function (MF), and cellular component (CC) were filtered by considering p-value < 0.05 and FDR < 0.05.

**Figure 4:**
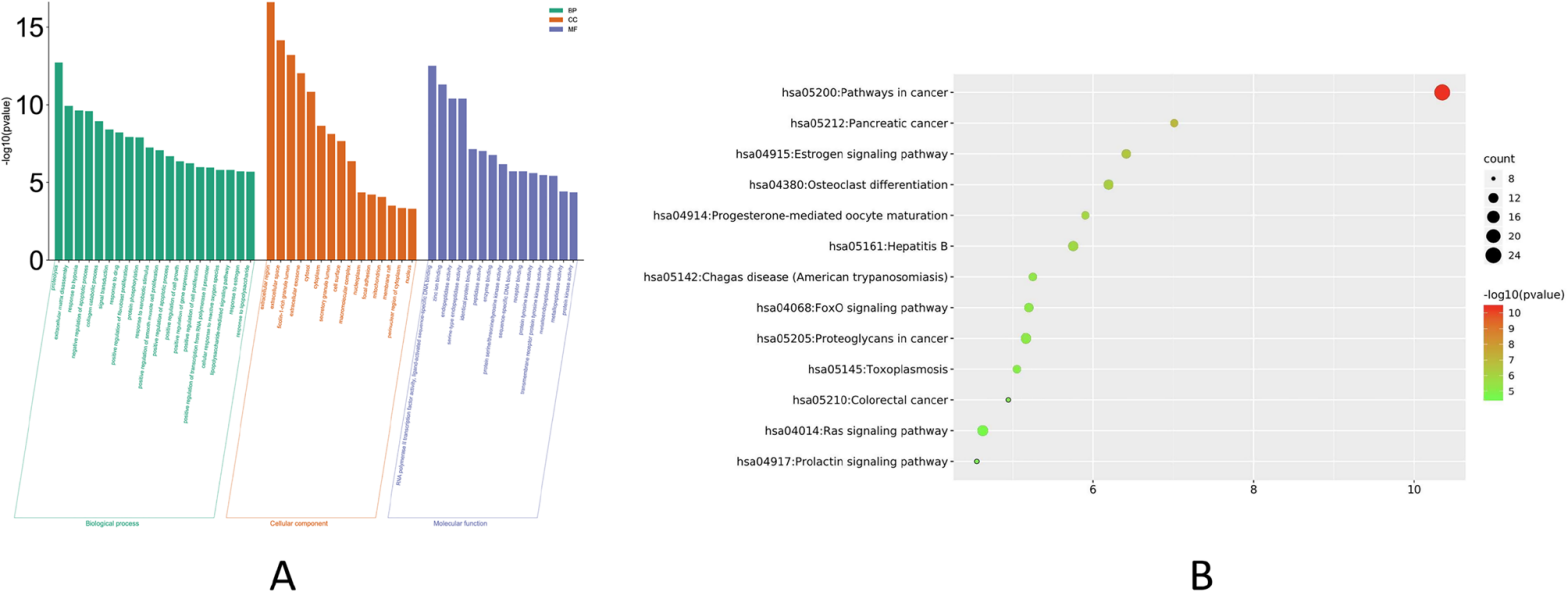
Functional enrichment analysis. (**A**) Enrichment analysis of potential targets of the active components of AA in rheumatism. The analysis of the Gene Ontology (GO) terms for biological process (BP), cellular component (CC), and molecular function (MF) is shown. LogP is the log-value of the P-value. P < 0.05 is considered significant. The results of the enrichment analysis are shown by LogP to present results intuitively. The count represents the number of genes, (**B**) Bubble diagram of KEGG enrichment analysis. The y-axis represents pathway and the x-axis represents gene ratio (amount of genes enriched in the pathway/amount of all genes in the background gene set). The size and color of the bubble represent the number of genes enriched in pathway and enrichment significance, respectively.

As shown in Fig. 4A, BP was dominated by the proteolysis, extracellular matrix disassembly, response to hypoxia, negative regulation of apoptotic process and collagen catabolic process. CC was dominated by extracellular region, extracellular space, ficolin-1-rich granule lumen, extracellular exosome and cytosol. MF was dominated by zinc ion binding, the same protein binding, serine-type endopeptidase activity, drug binding and receptor binding. All the above GO entries played important roles in RA.

Thirteen pathways were selected in accordance with p < 0.05 and FDR < 0.05 (Fig. 4B) to clarify the key pathways of 105 potential targets for rheumatism treatment. These pathways included those involved in cancer (hsa05200), pancreatic cancer (hsa05212), estrogen signaling pathway (hsa04915), osteoclast differentiation (hsa04380), progesterone-mediated oocyte maturation (hsa04914), hepatitis B (hsa05161), Chagas disease (American trypanosomiasis, hsa05142), FoxO signaling pathway (hsa04068), protein-polysaccharide in cancer (hsa05205), toxoplasmosis (hsa05145), colorectal cancer (hsa05210), Ras signal pathway (hsa04014), and prolactin signal pathway (hsa04917).

**Figure 5:**
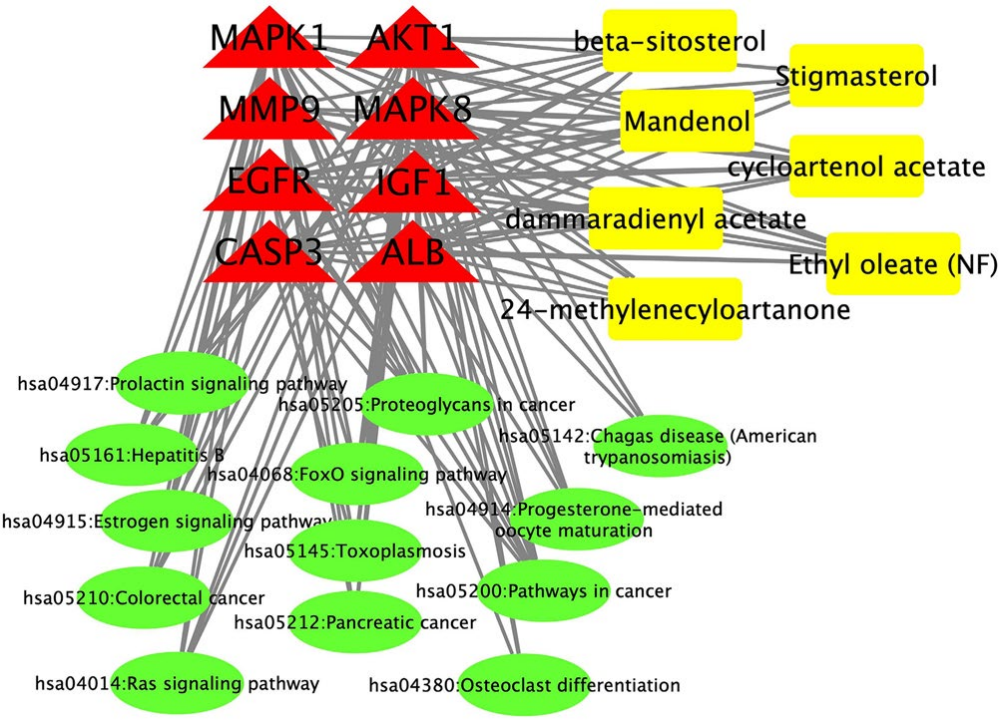
The compound target network associated with AA in rheumatism was produced by Cytoscape (Version, 3.8) (http://cytoscape.org/). The rectangle, triangle, and circle represent the component, target, and rheumatism-related pathway.

GO was annotated, and the response pathway was analyzed. By importing the effective target proteins, chemical components, and pathways into Cytoscape software, the compound-target-pathway network diagrams were set up (Fig. 5).

### Molecular docking results

**Figure 6:**
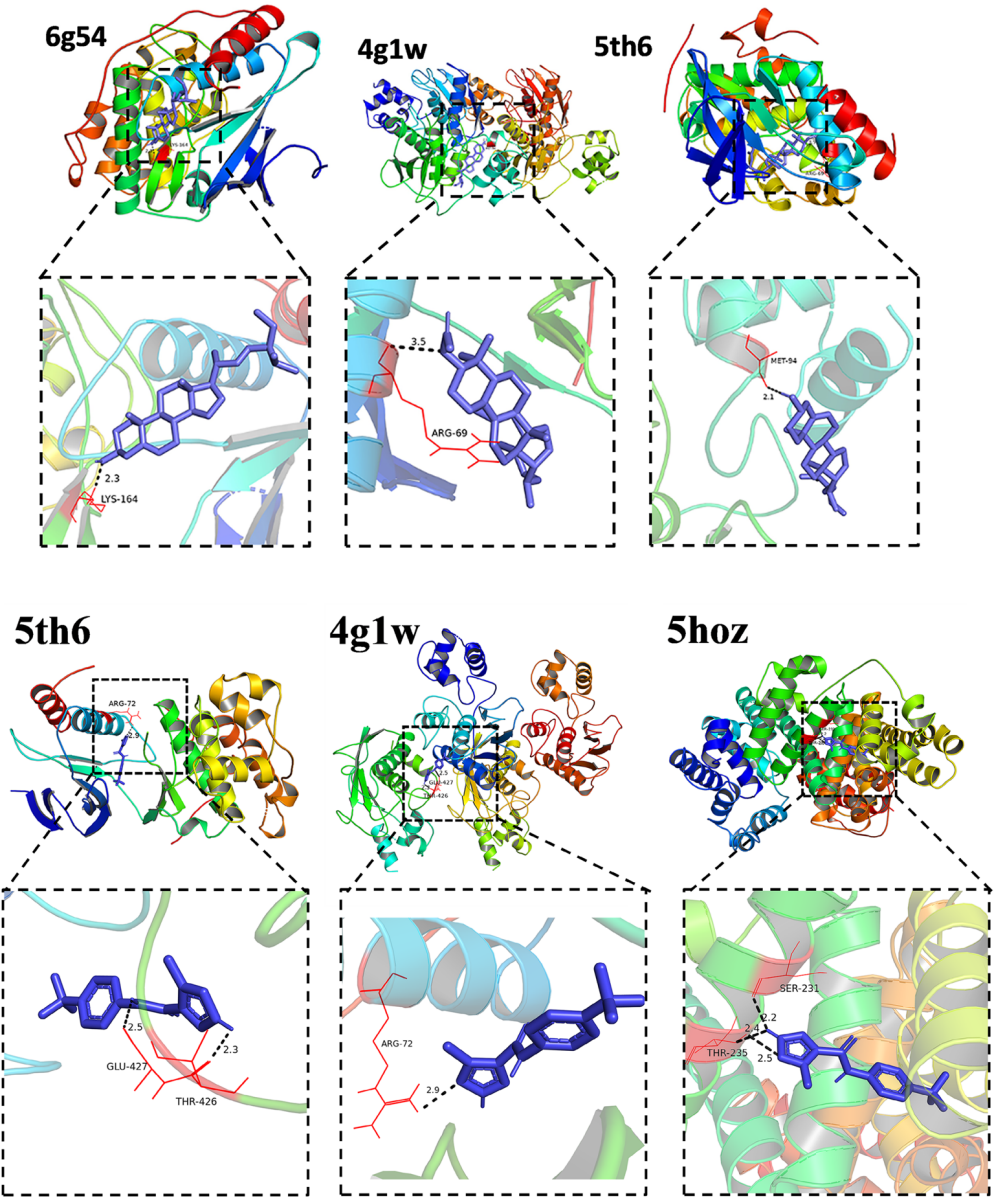
The docking posture and interactions between Strigmasterol and 6G54, Cycloartenol Acetate and 4G1W, Dammaradienyl acetate and 5TH6, Leflunomide and 5th6, Leflunomide and 4g1w, Leflunomide and 5hoz.

Taking the binding energy ≤ − 5.0 kJ/mol as the standard, the molecular docking verification of key active components and key targets was carried out. The results show that the affinity of the two docking sites is less than that of − 5.0 kJ/mol, which indicates that there is a good binding activity between the key active components and key targets, proving that the prediction of this study is more reliable, as shown in Table 2. The molecular docking display is illustrated in Fig. 6.

**Table 2:** Binding energy between main active components and core targets.

Molecular name	Hub gene binding energy(kcal/mol)				
	ALB	AKT1	MAPK1	MAPK8	MMP9
Stigmasterol	− 9.2	− 6.9	− 7.4	− 7.8	− 8.1
Cycloartenol acetate	− 9.4	− 6.3	− 7.1	− 8.3	− 7.5
Dammaradienyl acetate	− 7.6	− 5.7	− 7.6	− 5.1	− 6.8
Beta-sitosterol	− 8.6	− 6.4	− 7.1	− 5.9	− 7.1
Cyclophosphamide	− 4.9	− 4.6	− 4.6	− 4.7	− 4.6
Leflunomide	− 8.5	− 6.4	− 6.5	− 7.0	− 6.9

## Discussion

RA is a chronic inflammatory and autoimmune disease that is distinguished by the excessive proliferation of synovium and inflammatory cell infiltration, causing the chronic inflammation of joints and secondary erosion of cartilage and bone. The main causes of the disease are immune response, infection, and endocrine disorders. In the present study, we propose a network pharmacology method that combines the strategy of component prediction and target path analysis. This method is used to explore the potential regulatory effects of AA on inflammation, immune system, and angiogenesis, providing a new perspective for the treatment of RA. The potential cooperation mechanism of EEAA is predicted and analyzed from the point of view of biological network, and preliminary experimental validations are performed.

In this study, 135 active components of AA and 105 potential targets of AA in the treatment of RA were screened out. PPI network identified ALB, AKT1, MAPK1, MAPK8, MMP9, EGFR and CASP3 as key potential targets. The molecular docking results showed that the active ingredients had good binding activity with potential targets. KEGG results showed the top five pathways (cancer, pancreatic cancer, et al.) are directly or indirectly involved in the formation of RF. Based on the experimental results and a number of articles on the therapy of RA by AA, the authors concluded that AA plays an important role in the treatment of RA by regulating the proliferation and differentiation of bone cells and osteoclasts and immune-inflammatory response.

The destruction of articular cartilage and bone is the main cause of disability. In recent years, a large number of related research demonstrated that osteoclasts could be the key to the pathological process of bone destruction in RA. The main active ingredient beta-sitosterol in AA can regulate the proliferation and differentiation of osteocytes and osteoclasts, so as to achieve the effect of treating RA^25^. AKT1 and MAPK8, as hub genes, play a major role in osteoclast differentiation. AKT1, a unique signaling intermediate in osteoblasts, can regulate the differentiation of osteoblast and osteoclast at the same time. The targeted inhibition of AKT1 can result in effective bone acquisition, and related AKT1 and AKT2 have significant influence on the cell differentiation pathway^26^. The MAPK8 signal body is the target to prevent joint destruction^27^. MAPK is a collection of evolutionarily conserved serine/threonine protein kinases, which are activated by certain extracellular stimuli and may startup the signal transmition from cell membrane to nuclear. The activation of the MAPK pathway can induce the expression of heat shock protein (which has the function of regulating cell proliferation) in osteoblasts, thus promoting the formation of osteoblasts. KEGG pathway analysis demonstrates that osteoclast differentiation and Ras signal pathway are involved in the formation of RA. Therefore, AA may serve a purpose in the treatment of RA by regulating the proliferation and differentiation of osteoclasts and osteoclasts.

RA is a chronic inflammatory and autoimmune disease characterized by excessive synovial proliferation and inflammatory cell infiltration, resulting in chronic inflammation of joints and resultant erosion of cartilage and bone. Therefore, AA plays an important role in the treatment of RA by regulating immune-inflammatory response. The main active ingredient stigmasterol in AA can regulate the expression of inflammatory factors, thus achieving anti-inflammatory effect^28^. In addition, MAPK8 is also called JNK, and its activation in RA synovium can mediate articular damage in rats with adjuvant-induced arthritis^29^. KEGG results showed that FOXO signaling pathway is directly associated with the formation of RA. FOXO3a can mediate TNF-α through PI3K/AKT signaling pathway to inhibit the proliferation and invasion of trophoblast cells and promote their apoptosis, thus being expected to result in inflammation in the body^30^. In vitro experiment, AA could significantly down regulate the level of IL-17, IL-1βand TNF-α in RA rats^31^. Therefore, the authors suggest that AA achieves its therapeutic effect on rheumatoid arthritis mainly by regulating the immune-inflammatory response. Therefore, MAPKs and Akt signal pathway are promising targets for RA therapy, and the research and development of corresponding inhibitors for both pathways are hot spots in the research and development of RA therapeutic drugs. These results demonstrated the complicated relationship among compounds, targets, and pathways of AA in rheumatism^32^.

However, this research has the following three limitations: (1) all the prediction results were achieved by analysis based on limited online databases, which are kept updating. (2) The composition of AA is so complex that some compounds not identified are likely not involved in the analysis and discussion. (3) The future study should focus on verification experiments which help to explain the prediction results of this study.

## Conclusion

TCM usually plays an important role to prevent and treat various diseases. Therefore, in this paper, the network pharmacology method was used to screen RA-related targets and AA signaling pathways. Thus, the genomic space might be connected to the pharmacological space. In conclusion, we predicted the mechanism of action of EEAA in the treatment of RA by analyzing key targets and KEGG pathway. The methods applied to TCM should correspond to the synergistic mechanism.

## Data Availability

Data inquiries can be directed to the corresponding author.
